# Optimization of Preparation Technology and Evaluation of Antimicrobial Products by Glycerolysis of Black Soldier Fly (*Hermetia illucens*) Larvae Oil

**DOI:** 10.3390/insects16111163

**Published:** 2025-11-14

**Authors:** Kun Luo, Chen Chen, Jiaxin Liao, Junbo He, Yanxia Cong, Weinong Zhang

**Affiliations:** 1 Key Laboratory for Deep Processing of Major Grain and Oil, Ministry of Education, College of Food Science & Engineering, Wuhan Polytechnic University, Wuhan 430023, China; luokun524@163.com (K.L.); chchen@whpu.edu.cn (C.C.); xy522011@163.com (J.L.); junb112he@whpu.edu.cn (J.H.); congyanxia_l@163.com (Y.C.); 2 Hubei Key Laboratory for Processing and Transformation of Agricultural Products, Wuhan Polytechnic University, Wuhan 430023, China; 3 Engineering Research Center of Lipid-Based Fine Chemicals of Hubei Province, Wuhan 430023, China

**Keywords:** black soldier fly oil, glycerol monolaurate, response surface optimization, antimicrobial activity, methicillin-resistant *Staphylococcus aureus*

## Abstract

The growing problem of antibiotic resistance and associated side effects underscores the need for exploring novel therapeutic strategies. The utilization of insect resources is being investigated as one potential avenue in this context. The effective utilization of insect resources represents a promising pathway to this end. This study focuses on investigating the glycerolysis of black soldier fly (*Hermetia illucens*) larvae (BSFL) oil, which is rich in lauric acid, to optimize the production of antimicrobial monoglycerides. Response surface optimization yielded the following optimal conditions: 35.5 min, 219 °C, 0.72% sodium methoxide catalyst, and a 1:4 molar ratio of triglyceride to glycerol. Under these conditions, monoglycerides accounted for 55.86% of the product, specifically glycerol monolaurate, accounting for 29.47%; this mixture showed notable antimicrobial activity against *Staphylococcus aureus*, methicillin-resistant *Staphylococcus aureus* (MRSA), *Staphylococcus epidermidis*, *Escherichia coli*, and *Pseudomonas aeruginosa*. After purification via the solvent crystallization method, the monoglyceride content rose to 69.64%, while the glycerol monolaurate content increased to 35.24%, resulting in enhanced antimicrobial efficacy. Notably, monoglycerides were more effective against Gram-positive than Gram-negative bacteria, consistent with their known membrane-targeting specificity. Importantly, the potent activity against MRSA highlights the potential of these MAGs to combat antibiotic-resistant strains. These findings indicate that BSFL oil is a sustainable feedstock for producing antimicrobial agents with in vitro efficacy. This work supports the further investigation of MAGs derived from BSFL oil as potential candidates to complement existing antibiotics, particularly against resistant strains such as MRSA.

## Simple Summary

This study addresses the potential of using oil from black soldier fly larvae as a sustainable in vitro antimicrobial source that may complement combatting against antibiotic resistance. This study aimed to optimize the process of converting this insect oil into natural antimicrobial compounds called monoglycerides. Under the best conditions, the resulting product contained over 55% monoglycerides and showed strong activity against various bacteria, including methicillin-resistant *Staphylococcus aureus* (MRSA). Purification further increased both the monoglyceride content and the antimicrobial efficacy. The research concludes that black soldier fly larvae oil is an excellent raw material for producing effective natural antimicrobials, which provides a promising and eco-friendly starting point for further exploration as potential candidates to supplement existing strategies against antibiotic-resistant infections in food, medical, and pharmaceutical applications.

## 1. Introduction

Gram-positive bacterial infections, notably *Staphylococcus aureus (S. aureus)* and *Staphylococcus epidermidis (S. epidermidis)*, can manifest as local skin abscesses or progress to severe systemic infections with potentially fatal outcomes [1,2,3]. The emergence and spread of MRSA, in particular, pose a major challenge to global public health due to its multidrug resistance. The primary approach against bacterial infections continues to rely on conventional antibiotics (e.g., quinolones, aminoglycosides), which may result in adverse effects in patients, such as allergic reactions, hepatorenal toxicity, and neurotoxicity, and may also contribute to the development of further antibiotic resistance in pathogens [4,5,6,7]. Hence, there is a clear imperative to develop novel, eco-friendly, and safe natural antimicrobial agents. Many natural antimicrobials sourced from food or plants, such as monoglycerides (MAGs), which are commonly found in food emulsifiers like glycerol monolaurate (GML), exhibit high safety profiles and minimal side effects [8]. In contrast to traditional antibiotics that target specific bacterial biochemical pathways (e.g., protein synthesis, DNA replication), MAGs primarily function by physically disrupting bacterial cell membranes [9]. This makes it more challenging to induce bacterial resistance. MAG, as an attractive class of natural antimicrobials derivable from biomass resources, holds foreseeable potential based on in vitro evidence to serve as promising candidates for complementing or supplementing the current arsenal of conventional antibiotics after further evaluation.

MAGs can be synthesized from black soldier fly (*Hermetia illucens*) larvae (BSFL) oil. Unlike traditional oil sources, BSFL represent a valuable and eco-friendly biomass resource, as they thrive on organic waste such as kitchen refuse and possess an exceptionally high oil content (exceeding 45% on a dry weight basis) [10,11,12]. Notably, BSFL oil is rich in medium-chain fatty acids, especially lauric acid [13,14]. MAGs derived from this oil, primarily GML, have been extensively demonstrated to exhibit significantly greater antimicrobial potency than those sourced from common botanical origins [9,15,16]. Consequently, the synthesis of MAGs from BSFL oil leverages the unique advantages of this feedstock and also offers a synergistic enhancement in both environmental sustainability and functional efficacy.

**Figure 1:**
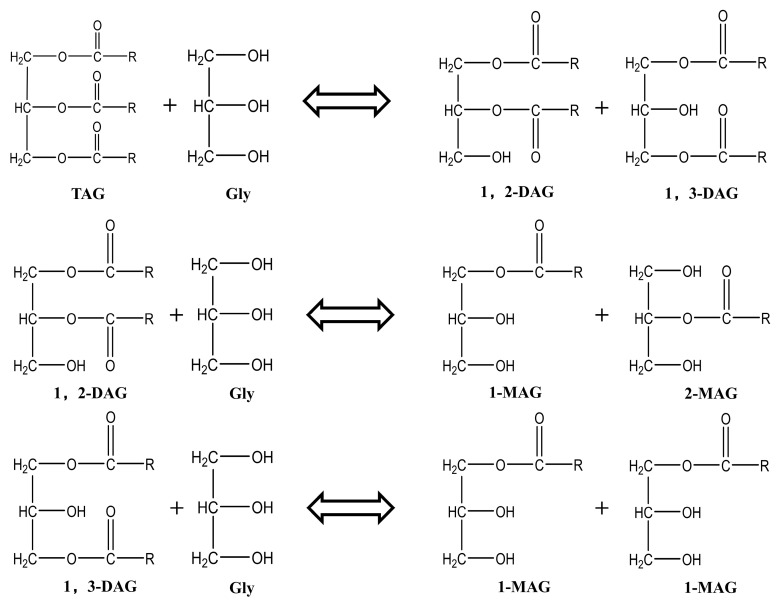
Glycerolysis reaction diagram. TAG triglyceride; Gly Glycerol; 1,2-DAG 1,2-diglyceride; 1,3-DAG 1,3-diglyceride; 1-MAG 1-monoglyceride; 2-MAG 2-monoglyceride.

Developing a cost-effective synthesis route is crucial for enhancing the antimicrobial efficacy of MAGs derived from insect oils. While multiple methods exist, glycerolysis of triglycerides is preferred due to its lower raw material costs compared to using free fatty acids or methyl esters [17,18]. This process, in which glycerol reacts with triglycerides to produce diglycerides and MAGs (with the former being further convertible), is outlined in Figure 1 [19,20]. Although this can be achieved through chemical or enzymatic methods, chemical catalysis using base catalysts is more suitable for industrial applications due to its greater cost-effectiveness. Critically, as neither diglycerides nor triglycerides of laurate exhibit antimicrobial properties [15], maximizing the MAG conversion rate is essential to ensure the efficacy of the final product. While current research on BSFL oil focuses on extraction and direct feed application [21,22,23], a significant gap remains in the production of potent antimicrobial MAGs from this specific resource.

This study identified an optimal glycerolysis process for BSFL oil, departing from conventional oil modification techniques used for oils such as soybean oil. It systematically optimized the key parameters and evaluated the antimicrobial properties of the resulting MAGs. The objective was to develop a comprehensive production pathway from a novel sustainable biomass resource (BSFL oil) to a potent natural antimicrobial substance. The ultimate aim is to explore its potential in providing safe and eco-friendly antimicrobial candidates for the food, medical, and pharmaceutical sectors.

## 2. Materials and Methods

### 2.1. Materials

Dry BSFL were provided by Inspro Science (Shenzhen) Co., Ltd. (Shenzhen, China); sodium hydroxide (≥96%), sodium methoxide (≥98%), analytically pure n-hexane, chloroform, glacial acetic acid, anhydrous ether, isopropanol, hydrochloric acid, methanol, glycerol and other analytically pure reagents were purchased from Shanghai National Pharmaceutical Chemical Reagent Co., Ltd. (Shanghai, China); chromatographically pure n-hexane, isopropanol and acetonitrile were purchased from Tianjin Kemiou Chemical Reagent Co., Ltd. (Tianjin, China). Analytical standards, including Monolaurin, Monopalmitin, Monoolein, and Dilaurin, were purchased from ANPEL Laboratory Supplies (Shanghai) Inc. (Shanghai, China). Lysogeny broth (LB) and LB agar were purchased from Qingdao Hope Bio-Technology Co., Ltd. (Qingdao, China). *Staphylococcus aureus* (BNCC186335) and *Escherichia coli* (*E. coli*, BNCC133264), Methicillin-resistant *Staphylococcus aureus* (MRSA, ATCC43300), *Staphylococcus epidermidis* (BNCC102555), and *Pseudomonas aeruginosa* (*P. aeruginosa*, BNCC337005) were purchased from Beina Chuanglian Biotechnology Co., Ltd. (Xinyang City, China).

### 2.2. Experimental Apparatus

Centrifugation of samples was performed using an H750R high-speed desktop refrigerated centrifuge (Hunan Xiangyi Laboratory Instrument Development Co., Ltd., Changsha, China). Constant-temperature stirring reactions were conducted in an SHJ-4AB magnetic stirring water bath (Changzhou Jintan Liangyou Instrument Co., Ltd., Changzhou, China). Analysis of volatile components was performed using a 7890A gas chromatograph (Agilent Technologies Co., Ltd., Santa Clara, CA, USA). High-temperature reactions were heated with a DF-101S oil bath (Shanghai Xiniuleibo Instrument Co., Ltd., Shanghai, China). Precise weighing of all reagents was achieved using an ME204/02 electronic balance (Mettler-Toledo Instrument Co., Ltd., Columbus, OH, USA). Solvent concentration and recovery were accomplished with a YRE-2000E rotary evaporator (Gongyi Yuhua Instrument Co., Ltd., Gongyi, China). High-performance liquid chromatography (HPLC) analysis was executed using a 1260 Infinity II HPLC system (Agilent Technologies Co., Ltd., Santa Clara, CA, USA) equipped with an ELSD600 evaporative light scattering detector (Shanghai Hengping Instrument Co., Ltd., Shanghai, China). Aseptic operations were performed inside an MCB-1300VAN medical clean bench (Zhongke Meiling Cryogenic Technology Co., Ltd., Hefei, China). Sterilization of experimental utensils was processed using an LX-B100L vertical automatic electrothermal pressure steam sterilizer (Hefei Huatai Medical Equipment Co., Ltd., Hefei, China). Constant-temperature shaking incubation was conducted in an HNY-100B intelligent constant temperature oscillator (Tianjin Ounuo Instrument Co., Ltd., Tianjin, China). Microbial or cell culture was carried out in an SPX-100 biochemical incubator (Shandong Laibo Instrument Co., Ltd., Jinan, China). Microplate absorbance detection was measured using a SPARK multifunctional microplate reader (Tecan Austria GmbH, Grödig, Austria).

### 2.3. Detection Method

The acid value and peroxide value were determined following AOCS official methods Cd 8d-90 and Cd 8d-53, respectively.

The fatty acid composition was analyzed according to AOCS Ce 2-66 (AOCS, 2017). Briefly, approximately 0.1 g of the oil sample was precisely weighed and transferred into a 10 mL stoppered glass tube. After the addition of 2 mL of 0.5 mol/L sodium hydroxide-methanol solution, the mixture was vortexed thoroughly and reacted in a 65 °C water bath for 30 min. This was followed by the addition of 2 mL of boron trifluoride-methanol solution and heating at 65 °C for 3 min. After cooling, 2 mL of n-hexane was added, and the mixture was vortexed again. Once the layers had separated, the supernatant was filtered through a 0.22 μm organic filter membrane for gas chromatographic analysis. Gas chromatography was performed using a Sopelcosp-2560 quartz capillary column (100 m × 0.25 mm × 0.2 mm, Merck KGaA, Darmstadt, Germany) with high-purity nitrogen as the carrier gas at a pressure of 31.31 psi and a split ratio of 20:1. The injection port and flame ion detector were maintained at 250 °C. The column temperature was initially set at 100 °C for 4 min, then ramped up to 230 °C at a rate of 3 °C/min, where it was held for 20 min. The total analysis time was 67.33 min. Fatty acid quantitative method: The relative percentage composition of each fatty acid was determined using the peak area normalization method without an internal standard.

Analysis of MAGs by HPLC. Sample pretreatment: The sample (100 mg) was dissolved in 5 mL of isopropanol, followed by a 30-fold dilution. The resulting solution was then filtered through a 0.22 μm membrane prior to analysis. For the calibration curve, approximately 10 mg of the standard compound was dissolved and made up to volume in a 10 mL volumetric flask to prepare the stock solution. Subsequently, the mother solution was diluted to obtain standard solutions of varying concentrations for GML (0.0204, 0.0425, 0.0850, 0.1020, 0.1275 mg/mL), linoleic acid MAG (0.0214, 0.0446, 0.0892, 0.107, 0.1338 mg/mL), and palmitate MAG (0.0200, 0.0417, 0.0833, 0.1000, 0.1250 mg/mL). Following filtration through a 0.45 μm organic filter membrane, these solutions were stored for subsequent analysis. The peak areas corresponding to the different concentrations were utilized for the construction of the standard curve. Chromatographic conditions included the use of a Venusil XBP C18 liquid chromatographic column (4.6 × 250 mm, 5 μm, Agela Technologies Co., Ltd., Tianjin, China), with a mobile phase consisting of acetonitrile-isopropanol (80:20) at a flow rate of 1 mL/min. A 20 μL injection volume was utilized, with a column temperature of 35 °C. The ELSD parameters were set at a drift tube temperature of 60 °C and a nitrogen flow rate of 2.0 L/min. Refer to Table 1 for the elution procedure. The content of MAGs was quantified using an external standard method.

Analysis of MAGs by Thin-layer chromatography. Dissolve 100 μL of the sample in 3 mL of a 1:1 (*v*/*v*) mixture of n-hexane and diethyl ether. Subsequently, apply approximately 3 μL of the resulting solution onto a silica gel G thin-layer plate using a glass capillary. The plate is then developed using a solvent system comprising n-hexane, diethyl ether, and glacial acetic acid in a 45:25:1 (*v*/*v*) ratio. Following development, remove the plate, evaporate the solvent, and subject it to iodine vapor for visualization of color development.

### 2.4. Preparation of BSFL

The oil from BSFL was extracted using the solvent extraction method. A total of 800 g of dried BSFL powder was weighed and mixed with n-hexane at a solid-to-liquid ratio of 1:2 (*w*/*v*). The mixture was then leached at 50 °C for 20 min, followed by suction filtration. After filtration, 1600 mL of fresh n-hexane was added to the residue, and the leaching process was repeated twice under the same conditions. The filtrates from all three cycles were combined and concentrated by vacuum rotary evaporation to obtain BSFL oil.

The BSFL oil was refined using hydration degumming and alkali refining deacidification methods. For degumming, a measured amount of crude oil was placed in a glass beaker on a magnetic stirring plate and heated to 75 °C. Deionized water equivalent to 3% of the oil weight was added at the same temperature and stirred for 30 min after stabilization. The mixture was then stirred at room temperature for 10 min, followed by centrifugation at 5000 r/min for 10 min to separate the degummed oil layer. Subsequently, for deacidification, the degummed oil was placed in a conical flask and heated in a water bath at 70 °C. An alkali solution, comprising 10% of the theoretical alkali amount with an excess of 25%, was added and stirred for 50 min until deacidification completion. To eliminate residual soap and free alkali, the oil was washed twice with water equivalent to 15% of the oil weight: first with light salt water and then with deionized water. After each wash, centrifugation at 5000 r/min for 10 min was performed to obtain the purified oil for further use.

### 2.5. Glycerolysis Single-Factor Experimental Design

A 10 g BSFL oil was combined with a specific molar ratio of glycerol (1:2, 1:3, 1:4, 1:5, or 1:6) in a 25 mL round-bottom flask. A precise amount of sodium methoxide catalyst (0.2%, 0.45%, 0.7%, 0.95%, or 1.2% of the BSFL oil mass) was then added to the mixture. The reactor was evacuated, purged with nitrogen, and heated in an oil bath at a predetermined temperature (170, 190, 210, 230, or 250 °C) with magnetic stirring. After a specified reaction time (15, 30, 45, 60, or 75 min), the reaction was quenched by the addition of citric acid. The mixture was allowed to stand for 10 min, after which the upper oil layer was collected for subsequent analysis.

### 2.6. Response Surface Optimization

Based on the results of the single-factor experiments, four critical factors—reaction time (A), reaction temperature (B), catalyst amount (C), and substrate molar ratio (D)—were selected as independent variables. The monoglyceride (MAG) content was defined as the response variable. A four-factor, three-level Box–Behnken Design (BBD) was employed to design the experiments and fit a second-order polynomial model. This design resulted in a total of 29 experimental runs, which were executed in a randomized order to minimize the effects of uncontrolled variables.

### 2.7. Purification of MAGs and Evaluation of Their Antimicrobial Effect

Isolation and purification of MAGs. MAGs were purified by the solvent crystallization method. The reaction product was weighed at 20 g, and hexane was added in a 1:10 (*w*/*v*) ratio. After fully mixing, it was crystallized at −30 °C. After suction filtration, the solid was taken and crystallized three times. After removing the solvent, it was reserved for use. The content of MAGs was determined by thin-layer chromatography and liquid chromatography.

Determination of antimicrobial rate. *S. aureus*, MRSA, *S. epidermidis*, *E. coli*, and *P. aeruginosa* were utilized as model microorganisms to assess the antimicrobial efficacy of the tested products. The bacteriostatic stock solution was prepared by dissolving 20 mg of the test substance in 0.5 mL of dimethyl sulfoxide (DMSO) and subsequently adding 9.5 mL of broth medium. Subsequently, 200 μL of bacterial solution (approximately 1 × 10^6^ cfu/mL) was added to individual disposable sterile shaking tubes, followed by the addition of 5 mL of antimicrobial solution diluted to 0.25 mg/mL. The mixtures were then incubated at 37 °C for 24 h. Next, 200 μL of the incubated solution was transferred to 96-well plates, and the absorbance (OD value) was measured at a wavelength of 595 nm using a multifunctional microplate reader. The antimicrobial rate was calculated based on the OD value of the control group, which contained a 0.625% DMSO solution in broth medium (identical to the concentration present in the antimicrobial solution).

Antimicrobial Rate (%)=(1−A/B)×100%

where *A*: OD value of the experimental group; *B*: OD value of the control group.

Evaluation of the antimicrobial effect of smearing. *S. aureus*, MRSA, *S. epidermidis*, *E. coli*, and *P. aeruginosa* were utilized as model microorganisms to assess the antimicrobial efficacy of the tested products. A 50 mg sample was weighed and dissolved in 2 mL of DMSO-LB solution (with a ratio of 1:3, *v*/*v*) to create an antimicrobial diluent. Subsequently, approximately 100 μL of a bacterial solution (containing around 1 × 10^6^ cfu/mL) was applied to LB agar plates using a sterile applicator. The plates were then partitioned into two sections: the left segment was treated with the antimicrobial diluent as the experimental group, while the right segment was treated with DMSO-LB solution as the control group. Bacterial growth was monitored after 24 h, and photographic documentation was conducted for record-keeping purposes.

### 2.8. Statistical Analysis

All experiments in this study were conducted with a minimum of three independent replicates, and data are presented as mean ± standard deviation. Statistical analyses were performed using specialized software as detailed below.

The optimization of the glycerolysis process was carried out using Response Surface Methodology (RSM). A Box–Behnken design (BBD) with four factors and three levels was employed, with the MAG content as the response variable. The experimental design, regression model fitting, analysis of variance (ANOVA), and generation of response surface plots were performed using Design Expert 13 software. Before analyzing variance, it is essential to perform model diagnostics and residual analysis. The repeated center points in the design represent independent experimental replicates (i.e., separately conducted reactions) rather than technical measurements of a single batch. The significance of the model and its terms was evaluated at a confidence level of *p* < 0.05.

For the antimicrobial activity assays, the inhibition rate data (percentage) were subjected to an arcsine square root transformation to stabilize variance and better meet the assumptions of parametric analysis. The significance of differences among the product groups against each bacterial strain was then assessed using one-way ANOVA on the transformed data. Each treatment was independently replicated three times (n = 3, representing three independent bacterial cultures prepared on different days, i.e., biological replicates). Post hoc comparisons were performed using Tukey’s HSD test to control the family-wise error rate, with statistical significance set at *p* < 0.05.

## 3. Results

### 3.1. Basic Index and Fatty Acid Composition of BSFL Oil

After degumming and deacidification, the acid value and peroxide value of the refined BSFL oil were (0.12 ± 0.01) mg KOH/g and (0.001 ± 0.000) g/100 g, respectively, indicating high purity suitable for subsequent glycerolysis. The fatty acid composition analysis revealed that lauric acid was the predominant medium-chain fatty acid, accounting for 24.00% of the total fatty acids (Table 2), highlighting the potential of BSFL oil as a feedstock for antimicrobial monoglyceride production.

### 3.2. Glycerolysis Single-Factor Experiment

**Figure 2:**
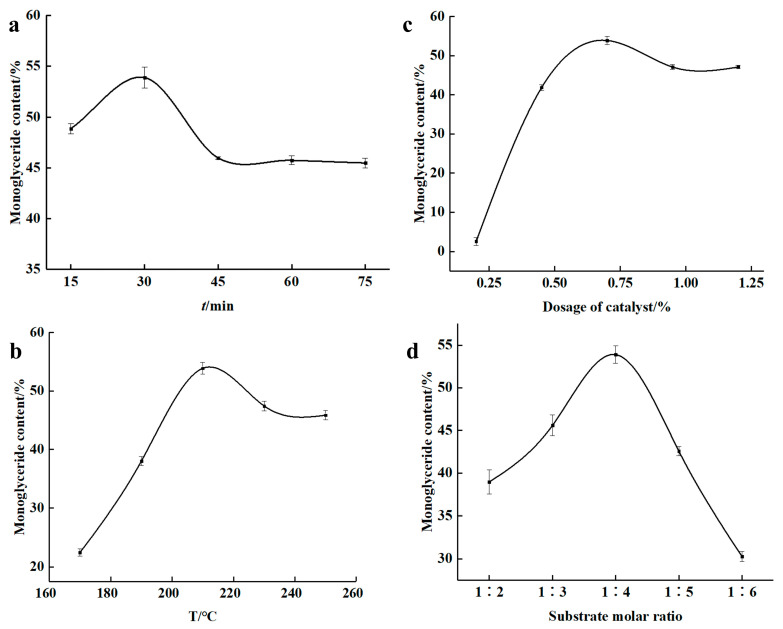
Influence of factors on the Monoglyceride (MAG) content. (**a**) time effect on the MAG content; (**b**) temperature effect on the MAG content; (**c**) catalyst amount effect on the MAG content; (**d**) substrate mole ratio effect on the MAG content.

The effects of reaction time, temperature, catalyst amount, and substrate molar ratio on MAG content were systematically investigated in a preliminary screening phase. For each factor investigated, the other parameters were fixed at baseline levels (30 min, 210 °C, 0.7% catalyst, 1:4 molar ratio). As shown in Figure 2a, the MAG content increased with time up to 30 min, after which it stabilized, suggesting a trend towards equilibrium. The highest MAG content was observed at 210 °C (Figure 2b), beyond which MAG content declined, likely due to thermal degradation. The catalyst amount exhibited a significant impact, with 0.7% sodium methoxide yielding the highest MAG content (Figure 2c). Similarly, a glycerol-to-oil molar ratio of 1:4 was found to be most effective (Figure 2d), beyond which excess glycerol diluted the catalyst and increased viscosity, thereby impairing reaction efficiency.

### 3.3. Response Surface Optimization

Based on the findings from the single-factor experiments (Section 3.2), which identified the approximate optimal ranges for each parameter, a Box–Behnken Design (BBD) was employed for response surface optimization. Four critical factors were selected as independent variables: reaction time (A), reaction temperature (B), catalyst amount (C), and substrate molar ratio (D). The specific levels for each factor, as shown in Table 3, were chosen centered around their individual optimal points: 30 min for time, 210 °C for temperature, 0.70% for catalyst amount, and a 1:4 molar ratio. The monoglyceride (MAG) content was defined as the response variable to systematically investigate the interaction effects between these parameters and identify the global optimum conditions.

Table 4 presents the response surface test design and outcomes. From the data comprising 29 test sets in Table 4, the regression equations for the MAG content (Y) in relation to reaction time (A), reaction temperature (B), catalyst amount (C), and substrate molar ratio (D) were derived as follows: Y = 52.81 + 6.58A + 14.58B + 3.68C − 2.03D − 3.61AB − 3.03AC + 1.44AD − 5.97BC + 2.26BD + 2.12CD − 7.01A^2^ − 15.86B^2^ − 6.9C^2^ − 6.44D^2^.

Prior to ANOVA, model diagnostics were performed. The residuals were examined and showed no obvious patterns against predicted values, and the constant variance assumption was reasonably met. The normality of residuals was assessed and found to be acceptable, supporting the use of ANOVA. The test results were analyzed, and the regression model ANOVA results are presented in Table 5. The model exhibited high significance with a *p*-value < 0.0001, indicating a strong fit. The non-significant *p*-value for Lack of Fit (0.1243, >0.05) suggests that the model is adequate and no significant lack of fit was detected relative to the pure error. The coefficient of determination (R^2^) was 0.9809, indicating that the model explains over 98% of the variation. Additionally, the high adjusted R^2^ (0.9617) and the reasonable agreement with the predictive R^2^ (0.8981) indicate that the model exhibits a good fit to the experimental data with a low risk of overfitting, despite the number of terms relative to the runs.

To further validate the model’s adequacy, diagnostic checks were performed. The variance inflation factor (VIF) for all model terms was less than 5, indicating no significant multicollinearity. Additionally, analysis of the residuals showed no obvious patterns and an approximate normal distribution, confirming that the underlying assumptions of the ANOVA were satisfied.

### 3.4. Response Surface Interaction Analysis

**Figure 3:**
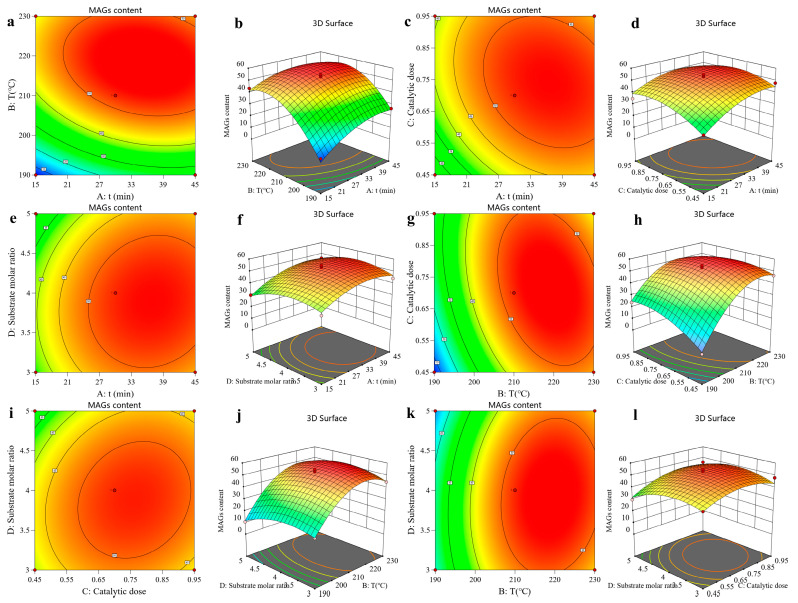
The effect of time, temperature, catalytic dose, and substrate molar ratio on the Monoglyceride (MAG) content. (**a**) and (**b**) show the contour and three-dimensional response surface plots, respectively, depicting the effects of reaction time and temperature on the MAG content. (**c**) and (**d**) show the contour and three-dimensional response surface plots, respectively, for the effects of reaction time and catalyst dosage on the MAG content. (**e**) and (**f**) show the contour and three-dimensional response surface plots, respectively, for the effects of reaction time and substrate molar ratio on the MAG content. (**g**) and (**h**) show the contour and three-dimensional response surface plots, respectively, depicting the effects of temperature and catalyst dosage on the MAG content. (**i**) and (**j**) show the contour and three-dimensional response surface plots, respectively, for the effects of temperature and substrate molar ratio on the MAG content. (**k**) and (**l**) show the contour and three-dimensional response surface plots, respectively, for the effects of catalyst dosage and substrate molar ratio on the MAG content.

The shape of the contour lines indicates the strength of the interaction effects. Circular contours denote nonsignificant interactions between two factors, whereas elliptical contours signify significant interactions. Analysis of Figure 3 reveals elliptical contour plots for reaction time and reaction temperature, reaction time and catalyst amount, and reaction temperature and catalyst amount, indicating significant interactions between these factors. Collectively, these statistically significant findings support that the developed model adequately describes the relationships between the process parameters and the MAG content in the glycerolysis process and exhibits a good fit to the experimental data.

Regression coefficient significance tests indicated that the linear terms for reaction time (A), temperature (B), and catalyst dosage (C) were highly significant, while the substrate molar ratio (D) was also a significant factor. The corresponding F-values demonstrated the relative impact of each factor on the MAG content, revealing the following order of influence: B > A > C > D. This confirms that individual variations in these parameters systematically and substantially influence the MAG content, validating the trends observed in the single-factor experiments. Furthermore, the highly significant quadratic terms (A^2^, B^2^, C^2^, D^2^) indicate that the relationship between each factor and the MAG content is nonlinear, with a distinct optimum beyond which the yield declines. Significant interaction effects, particularly the highly significant BC (temperature × catalyst) interaction, reveal that the optimal level of one factor is dependent on the level of another, underscoring the complex interdependence of the process parameters. Collectively, these statistically significant findings confirm that the developed model robustly captures the complex dynamics of the glycerolysis process and exhibits excellent goodness-of-fit.

### 3.5. Confirmation and Validation of the Optimum Extraction Process

The model analysis revealed the optimal conditions for producing MAGs through the glycerolysis of BSFL oil: a reaction time of 35.5597 min, a reaction temperature of 218.596 °C, a catalyst amount of 0.7173%, and a substrate mole ratio of 1:4.0379, resulting in the MAG content of 56.95%. To validate these optimal conditions while considering practical feasibility, adjustments were made: a reaction time of 35.50 min, a reaction temperature of 219 °C, a catalyst amount of 0.72 wt%, and a substrate molar ratio of 1:4. Three replicate experiments were conducted based on the revised parameters, yielding the MAG content of (55.86 ± 2.60)%, with MAG laurate constituting (16.47 ± 0.75)% of the total MAG content, representing (29.46 ± 0.04)% of the overall MAG content, closely aligning with the predicted value of 56.95%. The close agreement between the predicted and experimental values validates the model’s robustness and confirms its satisfactory predictive accuracy for the glycerolysis process within the studied design space.

### 3.6. Purification of Reaction Products

**Figure 4:**
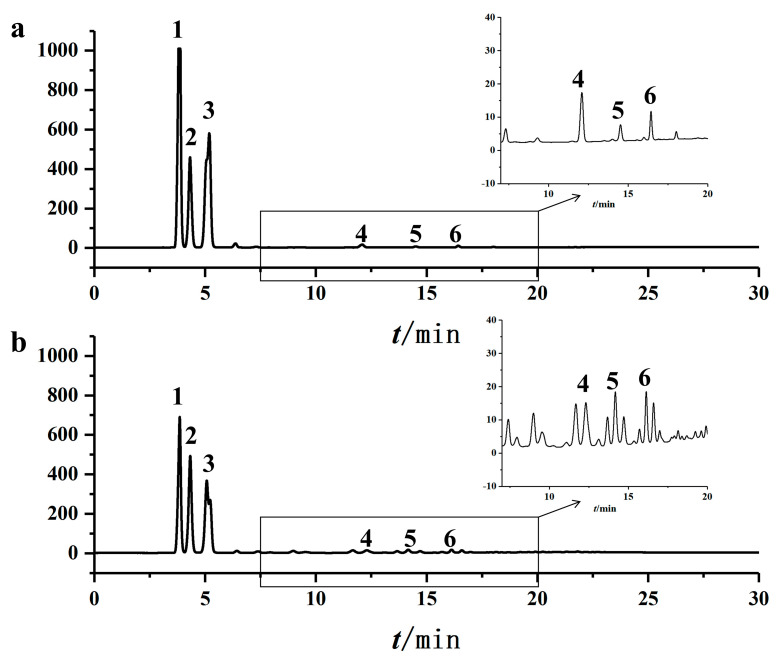
The HPLC chromatograms before and after the purification of Monoglycerides (MAGs). (**a**) after MAGs purification; (**b**) before MAGs purification. In the chromatograms, peaks 1, 2, and 3 correspond to MAG (C12), MAG (C18), and MAG (C16); peaks 4, 5, and 6 might be diglycerols, respectively.

**Figure 5:**
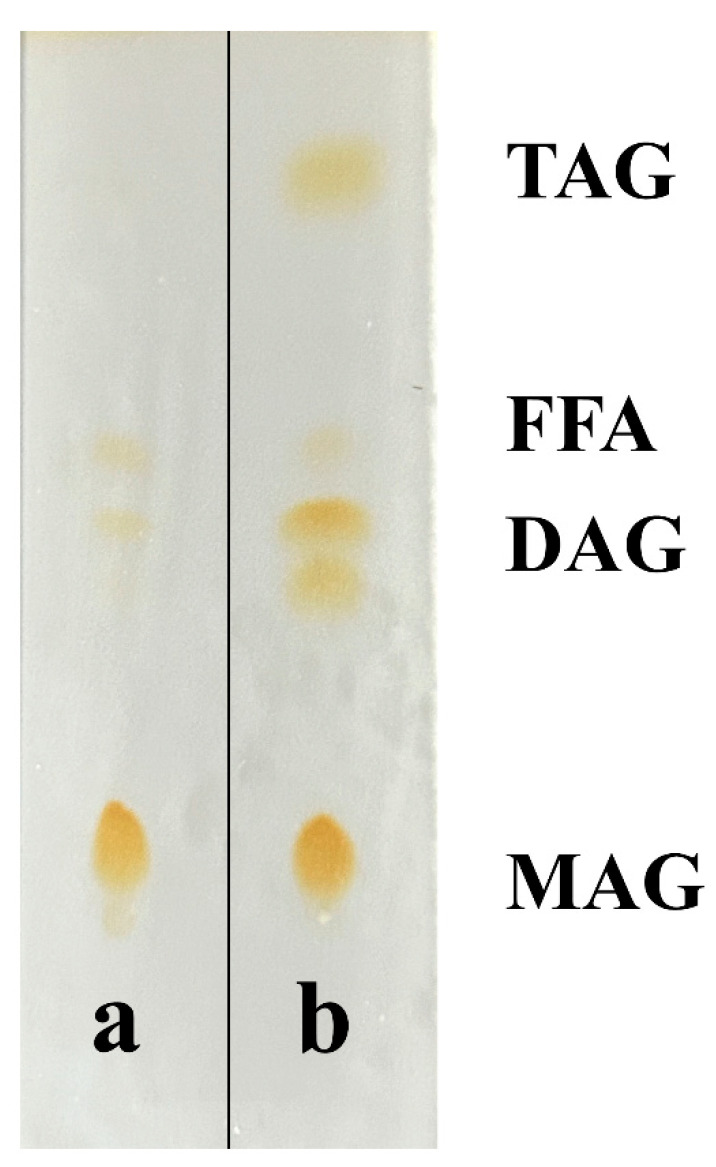
TLC before and after purification of Monoglycerides (MAGs). (**a**) after MAGs purification; (**b**) before MAGs purification. TAG: Triglyceride; FFA: Fatty acid; DAG: diglycerol; Monoglyceride (MAG).

Purification via solvent crystallization significantly enhanced the MAG content from 55.86% to 69.64 ± 0.05%, with glycerol monolaurate (GML) constituting 24.54 ± 1.56% of the total product, equivalent to 35.24 ± 2.28% of the total MAGs. HPLC and TLC analyses confirmed the effective removal of triglycerides and diglycerides (Figure 4 and Figure 5).

### 3.7. Antimicrobial Effect of MAGs

**Figure 6:**
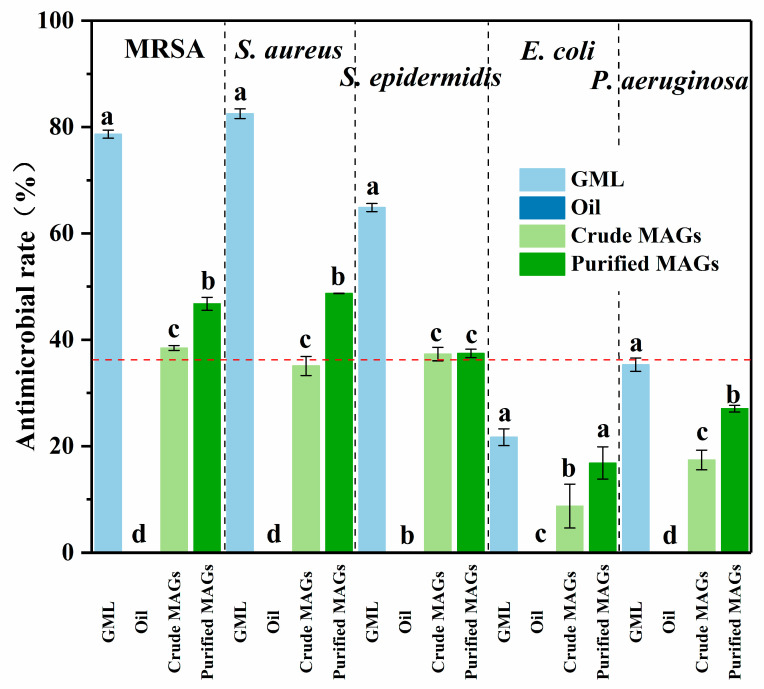
The antimicrobial efficacy of the product against four bacterial strains at a concentration of 0.25 mg/mL. The red dotted line indicates an antimicrobial rate of 36%. ANOVA revealed that the antimicrobial effects differed significantly among the groups with different monoglyceride contents for all bacterial strains (all *p* < 0.001). Different lowercase letters indicate significant differences (*p* < 0.001) based on one-way ANOVA of arcsine-transformed data followed by Tukey’s HSD test. (MRSA: methicillin-resistant *Staphylococcus aureus,* GML: 100% monoglyceride of laurate; BSFL oil: Black soldier fly oil without monoglycerides; Crude MAGs: Crude monoglycerides with ~50% monoglyceride content; Purified MAGs: Purified monoglycerides with ~70% monoglyceride content).

**Figure 7:**
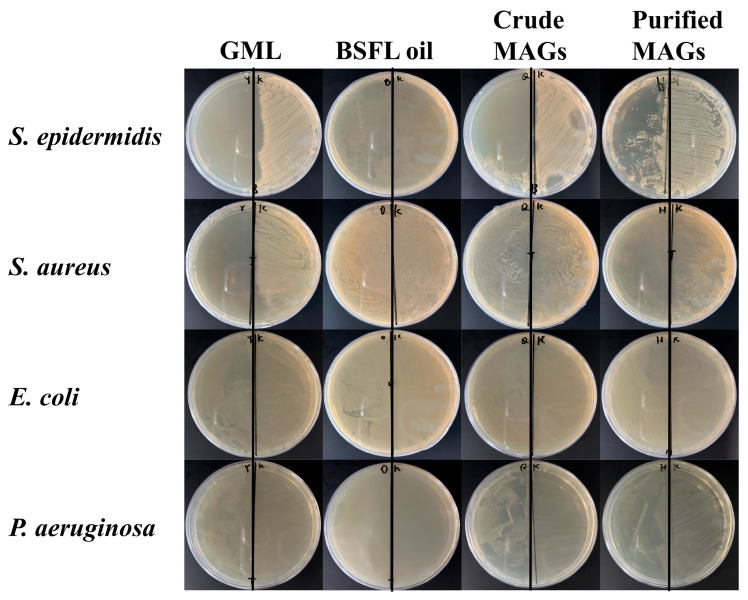
Antimicrobial effect of Monoglycerides against four bacteria. The left side of each graph received antimicrobial diluent, while the right side received a solution of Dimethyl sulfoxide and Luria–Bertani broth. GML:100% monoglyceride of laurate; BSFL oil: Black soldier fly oil without monoglycerides; Crude MAGs: Crude monoglycerides with almost 50% monoglyceride content; Purified MAGs: Purified monoglycerides with almost 70% monoglyceride content.

As shown in Figure 6, the crude BSFL oil showed no antimicrobial activity against any of the tested strains. In contrast, the glycerolysis-derived MAG product exhibited significant antibacterial effects. To evaluate statistical significance, a one-way ANOVA followed by Tukey’s HSD test was performed on the arcsine square root-transformed data. This analysis revealed a highly significant main effect of the treatment group for all bacterial strains (all *p* < 0.001; see Table 6 for F-values and degrees of freedom) and confirmed that the antimicrobial effects were significantly enhanced after purification. A clear difference in efficacy was observed between bacterial types; the MAGs were more effective against Gram-positive bacteria (*S. aureus*, MRSA, and *S*. *epidermidis*) than against Gram-negative ones (*E. coli* and *P. aeruginosa*). The antimicrobial activity was visually confirmed by the clear inhibition zones in the agar diffusion assay (Figure 7) and quantitatively supported by the reduction in bacterial growth area on the agar plates (Table 7). Collectively, these data from multiple methods provide consistent evidence for the in vitro antimicrobial efficacy of the MAGs under the tested conditions.

## 4. Discussion

This study successfully established an efficient pathway for converting BSFL oil into antimicrobial MAGs via glycerolysis. The high lauric acid content (24%) in BSFL oil provides a natural advantage for producing glycerol monolaurate (GML), a monoglyceride renowned for its potent antimicrobial properties [9,16]. Our optimization strategy, combining single-factor experiments and Response Surface Methodology (RSM), effectively identified the critical process parameters, and the resulting MAG product exhibited significant and selective antibacterial activity.

The single-factor experiments confirmed reaction temperature as the most critical parameter for MAG yield. The initial increase in yield with temperature, as reported by Luo et al. [19], is attributed to enhanced kinetics and molecular mobility. However, the decline beyond 210 °C aligns with established findings on thermal degradation and accelerated side reactions [18,24]. The role of sodium methoxide in generating glyceroxide anions was evident, mirroring its function in crude glycerol systems [25]. Yet, catalyst loadings above 0.7 wt% reduced yield, likely due to saponification and increased viscosity impairing mass transfer, a phenomenon noted in both model and crude glycerol systems [18,25]. The optimal glycerol-to-oil molar ratio of 4:1 represents a balance between driving the reaction equilibrium and avoiding negative effects of excessive glycerol, such as catalyst dilution, consistent with observations of glycerol saturation effects [26].

The RSM model provided a more comprehensive understanding, revealing significant interaction effects, particularly between temperature and catalyst amount. This interaction highlights the temperature-dependent nature of catalytic efficacy, a nuanced insight critical for process optimization that single-factor experiments overlook. Such synergistic effects between process parameters resonate with findings in hybrid catalytic systems [27]. The strong agreement between the predicted (56.95%) and experimental (55.86%) MAG yields under optimized conditions validates the model’s reliability and practical utility.

The selection of bacterial strains, including *S. aureus* and *E. coli* as benchmarks for Gram-positive and Gram-negative bacteria, respectively, and the addition of *S. epidermidis* (a predominant skin flora) and the highly resistant *P. aeruginosa*, was based on their representativeness, pathogenicity, and relevance to potential applications [9,28,29]. The stark difference in efficacy between BSFL oil (inactive) and the glycerolysis-derived MAGs unequivocally demonstrates that the antimicrobial activity is a direct result of the monoglycerides formed during the process, further confirming that residual DAG/TAG do not contribute to efficacy, as supported by literature and the enhancement of activity upon purification (Figure 4, Figure 5, Figure 6 and Figure 7) [15].

The superior efficacy against Gram-positive bacteria is consistent with the established membrane-targeting mechanism of MAGs [30]. Gram-positive bacteria possess a single, thick peptidoglycan layer that is susceptible to disruption by MAGs, which can inhibit peptidoglycan crosslinking, leading to loss of cell integrity. In contrast, the formidable barrier of the outer membrane in Gram-negative bacteria, comprising negatively charged lipopolysaccharides, effectively repels hydrophobic molecules like MAGs (e.g., monoolein and monopalmitin), thereby limiting their penetration [31,32]. The mechanism involves the spontaneous adsorption of MAGs to microbial membranes via their amphiphilic structure, leading to disruption of the phospholipid bilayer, increased permeability, efflux of intracellular components, and ultimately, cell lysis.

The potent in vitro activity of MAGs against MRSA underscores their potential efficacy against antibiotic-resistant strains in this context and warrants further investigation. As a major global health threat, MRSA exhibits resistance to multiple antibiotics; our findings suggest that MAGs may circumvent these conventional resistance mechanisms by directly targeting the bacterial membrane. This membrane-disrupting mode of action is hypothesized to pose a high barrier for the development of bacterial resistance, positioning MAGs as promising candidates for future research in combating antimicrobial resistance.

## 5. Conclusions

This study successfully established an optimized glycerolysis process for converting black soldier fly larvae (BSFL) oil into antimicrobial monoglycerides (MAGs). Under the determined optimal conditions (35.5 min, 219 °C, 0.72% sodium methoxide, 1:4 oil-to-glycerol ratio), a high MAG content of 55.86% was achieved. Subsequent purification via solvent crystallization significantly increased the MAG content to 69.64%, with a corresponding enhancement in antimicrobial efficacy. The resulting MAG product exhibited strong and selective in vitro activity against Gram-positive bacteria, including MRSA, underscoring its potential as a promising natural candidate in the fight against antibiotic-resistant bacteria. These findings underscore the value of BSFL oil as a sustainable feedstock for producing high-value antimicrobial agents and lay the groundwork for developing eco-friendly solutions for the food, cosmetic, and pharmaceutical industries. Future work should focus on developing purified MAG-based formulations, designing combination antimicrobial systems, and evaluating their safety and efficacy through in vitro and in vivo studies to facilitate practical application.

## Figures and Tables

**Table 1:** Gradient elution procedure for glyceride detection.

*t*/min	Acetonitrile/%	Isopropyl Alcohol/%	Flow Velocity (mL/min)
0	80	20	1
7	80	20	1
20	20	80	1
26	20	80	1
32	80	20	1
35	80	20	1

**Table 2:** Fatty acid composition.

Fatty Acid Species	Fatty Acid Content (%)
Lauric acid	24.00 ± 1.73
Myristic acid	5.00 ± 0.00
Palmitic acid	17.29 ± 0.38
Oleic acid	27.94 ± 0.86
Linoleic acid	14.19 ± 0.47
Linolenic acid	1.59 ± 0.06
Others	9.98 ± 0.05

Values are shown as mean ± standard deviation (SD). To reduce variability, focus on the effects of the components, and better simulate industrial application scenarios, we pooled BSFL from multiple batches and then extracted and analyzed the crude oil. The data is derived from three technical replicates (n = 3) conducted on the same batch of refined black soldier fly oil.

**Table 3:** Response surface optimization experimental design factors and levels.

Influence Factor	Level		
	−1	0	+1
Time (min)	15	30	45
Temp (°C)	190	210	230
Amount of catalyst (%)	0.45	0.70	0.95
Substrate molar ratio (black soldier fly larvae oil/glycerol)	1:3	1:4	1:5

**Table 4:** Experimental results of response surface optimization.

Run	Time	Temp	Amount of Catalyst	Substrate Molar Ratio	The Monoglyceride Content
1	30	210	0.95	1:3	47.89
2	30	210	0.70	1:4	50.78
3	45	210	0.70	1:5	45.88
4	30	210	0.70	1:4	51.61
5	15	210	0.70	1:3	34.15
6	15	210	0.70	1:5	30.07
7	45	190	0.70	1:4	26.44
8	30	210	0.45	1:3	40.13
9	30	210	0.95	1:5	45.85
10	30	230	0.70	1:3	44.53
11	30	230	0.45	1:4	46.50
12	30	230	0.95	1:4	42.23
13	30	190	0.95	1:4	23.95
14	45	210	0.45	1:4	47.82
15	30	190	0.70	1:5	10.77
16	30	210	0.70	1:4	54.95
17	45	230	0.70	1:4	47.41
18	45	210	0.70	1:3	44.19
19	45	210	0.95	1:4	44.14
20	30	210	0.45	1:5	29.59
21	30	230	0.70	1:5	44.35
22	15	230	0.70	1:4	43.46
23	30	210	0.70	1:4	52.93
24	15	210	0.45	1:4	26.39
25	30	190	0.45	1:4	4.34
26	15	210	0.95	1:4	34.83
27	15	190	0.70	1:4	8.04
28	30	210	0.70	1:4	53.78
29	30	190	0.70	1:3	20.00

Runs 2, 4, 16, 23, and 28 are center points representing independent experimental replicates (separately conducted reactions).

**Table 5:** Regression model analysis results.

Source	Sum of Squares	df	Mean Square	F-Value	*p*-Value	
Model	5411.62	14	386.54	51.22	<0.0001	significant
A-*t*	519.29	1	519.29	68.81	<0.0001	
B-T	2550.33	1	2550.33	337.94	<0.0001	
C-mount of the catalyst	162.21	1	162.21	21.49	0.0004	
D-substrate molar ratio	49.53	1	49.53	6.56	0.0226	
AB	52.20	1	52.20	6.92	0.0198	
AC	36.72	1	36.72	4.87	0.0446	
AD	8.32	1	8.32	1.10	0.3114	
BC	142.56	1	142.56	18.89	0.0007	
BD	20.48	1	20.48	2.71	0.1218	
CD	18.06	1	18.06	2.39	0.1441	
A^2^	318.63	1	318.63	42.22	<0.0001	
B^2^	1631.35	1	1631.35	216.17	<0.0001	
C^2^	309.16	1	309.16	40.97	<0.0001	
D^2^	268.70	1	268.70	35.61	<0.0001	
Residual	105.65	14	7.55			
Lack of Fit	94.56	10	9.46	3.41	0.1243	not significant
Pure Error	11.10	4	2.77			
Cor Total	5517.28	28				

A, B, C, and D represent time, temperature, catalyst dosage, and substrate molar ratio, respectively.

**Table 6:** The F-values and degrees of freedom of the antimicrobial efficacy.

Bacterial Species	F	df	*p*
*S. aureus*	1378.176	3, 8	<0.001
MRSA	892.905	3, 8	<0.001
*S. epidermidis*	956.051	3, 8	<0.001
*E. coli*	67.685	3, 8	<0.001
*P. aeruginosa*	674.490	3, 8	<0.001

MRSA: methicillin-resistant *Staphylococcus aureus.*

**Table 7:** The proportion of bacterial climbing boards.

Bacterial Species	GML (%)	BSFL Oil (%)	Crude MAGs (%)	Purified MAGs (%)
***S. aureus***	45.11	0.00	50.55	54.78
***S. epidermidis***	42.14	0.00	33.92	41.26
***E. coli***	93.99	0.00	89.42	95.17
***P. aeruginosa***	90.10	0.00	86.11	84.81

GML: 100% monoglyceride of laurate; BSFL oil: Black soldier fly oil without monoglycerides; Crude MAGs: Crude monoglycerides with ~50% monoglyceride content; Purified MAGs: Purified monoglycerides with ~70% monoglyceride content.

## Data Availability

The original contributions presented in this study are included in the article. Further inquiries can be directed to the corresponding author.

## Abbreviations

**ANOVA**	Analysis of Variance	**HPLC**	High-Performance Liquid Chromatography
**BSFL**	Black Soldier Fly Larvae	**MAG(s)**	Monoglyceride(s)
**DAG**	Diglyceride	**MRSA**	Methicillin-Resistant *Staphylococcus aureus*
**DMSO**	Dimethyl Sulfoxide	**OD**	Optical Density
**ELSD**	Evaporative Light Scattering Detector	**RSM**	Response Surface Methodology
**GML**	Glycerol Monolaurate	**TAG**	Triglyceride
**TLC**	Thin-Layer Chromatography	**VIF**	Variance Inflation Factor
